# Depletion of *tet2* results in age-dependent changes in DNA methylation and gene expression in a zebrafish model of myelodysplastic syndrome

**DOI:** 10.3389/frhem.2023.1235170

**Published:** 2023-09-14

**Authors:** Yaseswini Neelamraju, Evisa Gjini, Sagar Chhangawala, Hao Fan, Shuning He, Chang-Bin Jing, Ashley T. Nguyen, Subhash Prajapati, Caroline Sheridan, Yariv Houvras, Ari Melnick, A. Thomas Look, Francine E. Garrett-Bakelman

**Affiliations:** 1 Department of Biochemistry and Molecular Genetics, University of Virginia, Charlottesville, VA, United States; 2 Department of Pediatric Oncology, Dana-Farber Cancer Institute, Boston, MA, United States; 3 Weill Cornell Medicine, New York, NY, United States; 4 Harvard Medical School, Boston, MA, United States; 5 Division of Hematology and Medical Oncology, Department of Medicine, Weill Cornell Medicine, New York, NY, United States; 6 Department of Medicine, University of Virginia, Charlottesville, VA, United States; 7 University of Virginia Cancer Center, Charlottesville, VA, United States

**Keywords:** TET2, myelodysplastic syndrome, hematopoietic progenitors, zebrafish, DNA methylation

## Abstract

**Introduction:**

Myelodysplastic syndrome (MDS) is a heterogeneous group of clonal hematopoietic disorders characterized by ineffective hematopoiesis, cytopenias, and dysplasia. The gene encoding ten-eleven translocation 2 (*tet*2), a dioxygenase enzyme that catalyzes the conversion of 5-methylcytosine (5mC) to 5-hydroxymethylcytosine, is a recurrently mutated tumor suppressor gene in MDS and other myeloid malignancies. Previously, we reported a stable zebrafish line with a loss-of-function mutation in the *tet2* gene. The *tet2*^*m/m*^-mutant zebrafish developed a pre-MDS state with kidney marrow dysplasia, but normal circulating blood counts by 11 months of age and accompanying anemia, signifying the onset of MDS, by 24 months of age.

**Methods:**

In the current study, we collected progenitor cells from the kidney marrows of the adult *tet2*^*m/m*^ and *tet2*^*wt/wt*^ fish at 4 and 15 months of age and conducted enhanced reduced representation of bisulfite sequencing (ERRBS) and bulk RNA-seq to measure changes in DNA methylation and gene expression of hematopoietic stem and progenitor cells (HSPCs).

**Results and discussion:**

A global increase in DNA methylation of gene promoter regions and CpG islands was observed in *tet2*^*m/m*^ HSPCs at 4 months of age when compared with the wild type. Furthermore, hypermethylated genes were significantly enriched for targets of SUZ12 and the metal-response-element-binding transcription factor 2 (MTF2)—involved in the polycomb repressive complex 2 (PRC2). However, between 4 and 15 months of age, we observed a paradoxical global decrease in DNA methylation in *tet2*^*m/m*^ HSPCs. Gene expression analyses identified upregulation of genes associated with mTORC1 signaling and interferon gamma and alpha responses in *tet2*^*m/m*^ HSPCs at 4 months of age when compared with the wild type. Downregulated genes in HSPCs of *tet2*-mutant fish at 4 months of age were enriched for cell cycle regulation, heme metabolism, and interleukin 2 (IL2)/signal transducer and activator of transcription 5 (STAT5) signaling, possibly related to increased self-renewal and clonal advantage in HSPCs with *tet2* loss of function. Finally, there was an overall inverse correlation between overall increased promoter methylation and gene expression.

## Introduction

The ten-eleven translocation 2 gene *tet2*, a dioxygenase enzyme that can catalyze the conversion of 5-methylcytosine (5mC) to 5-hydroxymethylcytosine, is a recurrently mutated gene in hematological malignancies, including myelodysplastic syndrome (MDS) (1, 2). TET2 functions as a tumor suppressor and thus loss-of-function mutations are found in myeloid malignancies (3–5). Because TET2 catalyzes the first step in CpG demethylation, *tet2* mutations (*tet2*^*m/m*^) are thought to result in a failure to remove aberrant methylation, and thus would be expected to lead to a gain of DNA hypermethylation within the malignant myeloid clones, leading to the repression, or otherwise the aberrant expression, of genes important for myeloid differentiation (6).

To clarify the mechanisms through which the loss of *tet2* contributes to myeloid malignancies using an *in vivo* animal model, we created a stable zebrafish line with loss-of-function mutations in the *tet2* gene generated through zinc finger nuclease technology (7). The 4-bp deletion in *tet2* (ACAT; *tet2*^*m/m*^) led to stop codons in amino acid 1162 (AA1162), which in turn disrupt the catalytic activity of the hydroxylase. The *tet2*^*m/m*^ fish are viable and fertile, which allows the interrogation of *tet2* loss in hematopoietic cells throughout development, from larvae to adulthood. The *tet2*^*m/m*^-mutant zebrafish developed myelodysplastic features in the kidney marrow at the age of 11 months (7). Myelodysplasia, reflected by aberrant nuclear and cytoplasmic maturation in May–Grünwald–Giemsa-stained blood and kidney marrow smears, was observed in the erythroid, myeloid, and progenitor cell lineages of all of the *tet2* homozygous, and a subset of the heterozygous, mutant fish (7). In contrast, myelodysplasia was not observed in the kidney marrow smears of 11-month-old wild-type fish within the erythroid or myeloid cell lineages (7). The diagnosis of MDS requires both evidence of trilineage dysplasia in smears of the developing marrow cells and significantly deceased cell numbers of mature cells of at least one hematopoietic cell lineage in the peripheral blood. However, the peripheral blood counts were normal in 11-month-old *tet2*-mutant zebrafish, indicating that the process was limited to a pre-MDS stage (7). By 24 months of age, each of the *tet2*-mutant fish developed anemia, which, together with the marked dysplasia of the developing hematopoietic progenitors in the kidney marrow, formed the basis for an MDS diagnosis. The *tet2*^*m/m*^ zebrafish model, which uniformly develops a dysplastic hematopoietic disorder that progresses to MDS with anemia thus allows for an interrogation of the underlying mechanisms that cause progression from disordered myeloid cell differentiation to fully transformed MDS. We have recently used this zebrafish model to investigate the selective activity of small-molecule drugs in the killing of *tet2* mutants, compared with wild-type HSPCs, a chemical biology consequence of genomic mutations, which is analogous to the well-known genetic relationship called “synthetic lethality” (8, 9).

Using the *tet2*^*m/m*^ zebrafish, we sought to understand the effect of *tet2* loss on the CpG methylation state of the genome and gene expression patterns to identify potential molecular mechanisms that have an association with clonal expansion of the progenitor cells and disease progression in this disease model. Stem and progenitor cells, characterized by disordered growth and differentiation, play an important functional role in MDS pathogenesis and disease relapse. To capture events during disease progression to the pre-MDS state in this specific cell population, we collected progenitor cells from the kidney marrows of 4- and 15-month-old adult *tet2*^*m/m*^ and *tet2*^*wt/wt*^ fish and studied the changes in the DNA methylation levels associated with the promoters and CpG islands of specific genes.

## Materials and methods

### Zebrafish husbandry

Zebrafish are vertebrate animals, and all experiments and animal husbandry were conducted in accordance with the Dana-Farber Cancer Institute’s Institutional Animal Care and Use Committee (IACUC)-approved animal research protocol #02–107. The stocks of wild-type and mutant lines were maintained in accordance with a previously reported protocol (10).

### Zebrafish genetic manipulation

One-cell fertilized zebrafish embryos were injected with various amounts of mRNAs encoding zinc finger nucleases (ZFNs) harboring DD/RR or EL/KK variant *Fok*I nuclease domains, as previously described (7). Site-specific ZFN function was verified by PCR on genomic DNA (gDNA) using *tet2*-specific primers and then sequencing. The resulting *tet2*^*m/m*^ zebrafish line, which has been described and characterized previously (7–9), was used in this study.

### Genotyping

For adult stages, fish were individually genotyped using one forward (5′ ATCTCCAAGGTCTTGCAACCTA 3′) and one reverse (5′ ATACAAGCCCTCATCCACTGAT 3′) primer, as previously shown (7). The forward and reverse primers were located in the sixth and eighth exons of *tet2*, respectively. The PCR products, 668 bp in length, were sequenced with the forward primer.

### Cell suspension preparation and flow cytometry.

The cell suspension preparation and flow cytometry were conducted as previously described (7). In short, 4-month- and 15-month-old wild-type *tet2*^*wt/wt*^ and *tet2*^*m/m*^ fish were anesthetized. The anesthesia of the fish was carried out prior to kidney removal to harvest the kidney marrow. The fish were anesthetized by immersion in a small fish tank containing 200 mg/L of tricaine methanesulfonate dissolved in fish water. The working solution (200 mg/L of tricaine methanesulfonate dissolved in fish water) was made fresh by diluting a stock solution of tricaine methanesulfonate (4 mg/mL) in deionized water buffered with Tris-HCL to pH 7.0, which was stored in a freezer at −20°C. Tricaine methanesulfonate is an ester-type local anesthetic agent that acts systemically when absorbed through the gills, and thereafter is distributed throughout the body via the blood and acts on the peripheral and central nervous systems. After the fish were completely anesthetized, the kidney containing the whole kidney marrow was surgically removed. We then euthanized the fish by transferring them to a small fish tank immersed in an ice bath with a higher concentration of tricaine methanesulfonate (of 500 mg/L), which was prepared fresh by diluting the stock solution as previously described. The fish were kept in this euthanizing dose of tricaine methanesulfonate for at least 30 minutes on ice and then frozen in plastic bags at −20°C in a freezer designated as the morgue, prior to disposal by our Animal Resource Facility.

The removed kidneys were dissected and placed in ice-cold 0.9 × phosphate-buffered saline (PBS) containing 5% fetal calf serum (FCS). Single-cell suspensions were generated by aspiration, followed by mild teasing of the kidney on a 40-mm nylon mesh filter with a pipette tip. Progenitor cells were sorted using a JF Aria II Cell Sorter (BD Biosciences) with high forward scatter/side scatter (FSC/SSC), as previously described.

### Enhanced reduced representation of bisulfite sequencing

The enhanced reduced representation of bisulfite sequencing (ERRBS) was conducted as previously described (11, 12). Briefly, high-molecular-weight DNA was isolated from the progenitor cells using Qiagen’s AllPrep^™^ extraction kit, per the manufacturer’s instructions. The DNA was then digested with the *Msp*I restriction enzyme. Fragments were purified and subjected to end repair, A-tailing, and ligation of methylated Illumina adapters. This was followed by bisulfite conversion and PCR amplification. The libraries were sequenced on an Illumina 2500 using a 50-bp single-end read approach, per the manufacturer’s instructions. The sequencing data were aligned with Zv9 as the reference genome using our in-house processing pipeline (11). The CpG sites with at least 10× coverage were used for downstream analysis. The sequencing statistics are included in Supplementary Table 1A.

### Identifying high-variance CpGs

For each CpG, the interquartile range (IQR) across all samples was calculated using the “iqr” function in R (version 3.3.0; The R Foundation for Statistical Computing, Vienna, Austria). CpGs with an IQR greater than the 90th percentile were annotated as high-variance CpGs.

### Bulk RNA sequencing

The RNA was isolated from the progenitor cells using Qiagen’s AllPrep extraction kit, per the manufacturer’s instructions. RNA-seq libraries were prepared using TruSeq^™^ RNA-Seq by poly(A) enrichment (Illumina) and sequenced on a HiSeq^®^ 2000 system (Illumina) using a 50-bp single-end approach, per the manufacturer’s instructions. Sequencing statistics are included in Supplementary Table 1B.

### RNA-seq quality control and alignment

FASTQ files were checked for quality using FASTQC (version 0.10.1). The reads were then aligned using STAR aligner (version 2.3.0) (13) in single-end mode. After sorting and indexing, the aligned files were used with BEDTools (version 2.16.1) (14) and the Ensembl zebrafish transcriptome (Zv9) to generate read counts for genes for all samples. We calculated the normalized expression values using DESeq2 normalization. The normalized expression values were averaged across the two replicates in each of the wild-type and mutant categories. Standard deviation was used to calculate the bounds for error bars.

### Principal component analysis

Principal component analysis (PCA) was conducted using the “prcomp” function in R. The first two PCs were plotted using ggplot2 in R.

### Differential methylation analysis

The change in DNA CpG methylation between any given groups for a given region was calculated using methylKit (15). Regions with a minimum coverage of 10 reads and a maximum coverage greater than the 90th percentile of read counts were excluded from the analysis. Regions with an absolute methylation difference of at least 15% at a statistical significance of less than 0.05 were annotated as differentially methylated regions (DMRs).

### Differential gene expression analysis

Differential gene expression analysis of any two comparisons was conducted using DESeq2 (version 1.14.1) (16) package in R (version 3.3.0). The batch correction variable accounting for variation between replicates was added in the design equation. For each comparison, genes with greater than five reads across the samples were retained for analysis. Genes with an absolute value of log-fold change greater than 1 and a q-value less than 0.05 were annotated as differentially expressed genes (DEGs).

### Pathway enrichment analysis

To identify significantly enriched pathways for a given set of genes (either DEGs or DMRs), we converted the zebrafish gene names to their human orthologs using BioMart from the Ensembl database (v70). Overlap of the human orthologs with known pathways was assessed using “enrichR” in the R package (17–19). Significantly enriched pathways were identified at a *p*-value less than 0.05.

## Results

**FIGURE 1:**
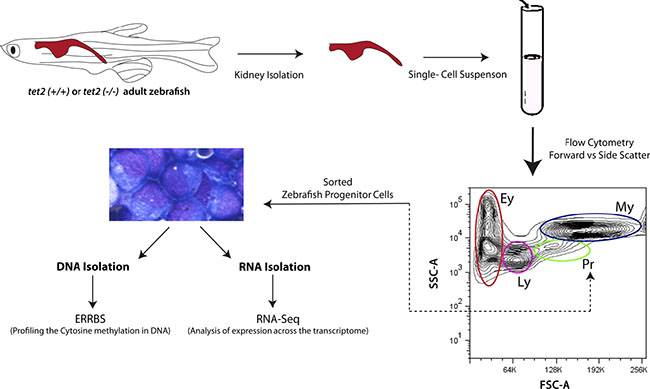
Schematic overview of studies of DNA methylation and RNA expression by zebrafish kidney marrow cells. The site of hematopoiesis in the adult zebrafish is in the head kidney (upper left of the diagram). Head kidney marrow cells from 4- and 15-month-old zebrafish were isolated and the progenitor cells were sorted by fluorescence-activated cell sorting based on their forward and side light scatter properties, as previously described (7). The sorted progenitor cells were then processed for DNA extraction using enhanced reduced representation of bisulfite sequencing to assess cytosine methylation. RNA was also extracted from an aliquot of the sorted progenitor cells for poly(A)+ RNA sequencing, as described in the Methods section. tet2, Ten-eleven translocation 2; ERRBS, Enhanced Reduced Representation of Bisulfite sequencing; Ey, erythrocytes; Ly, lymphocytes; Pr, progenitors; My, myelomonocytes.

**FIGURE 2:**
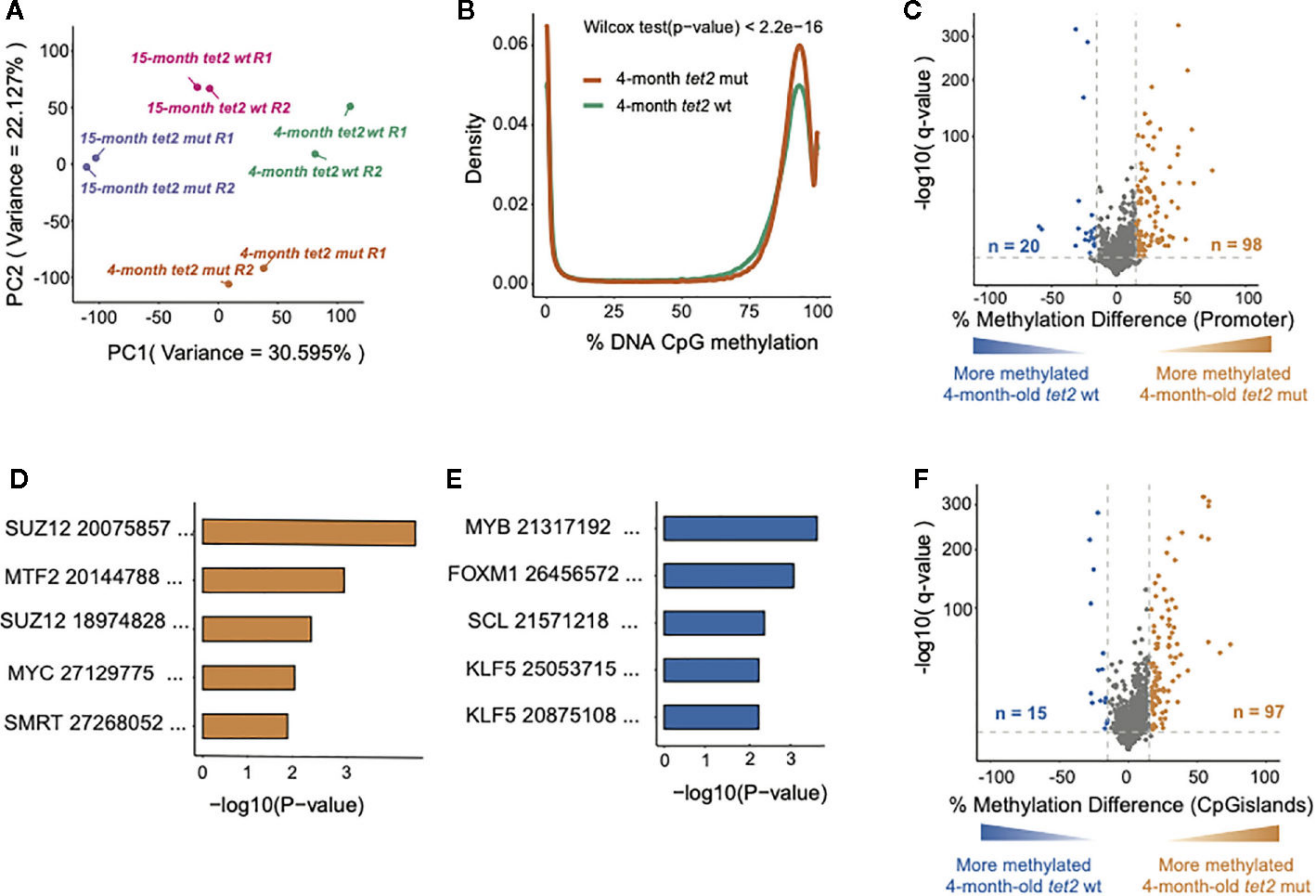
Loss of *tet2* associated with the gain of CpG methylation in HSPCs of 4-month-old. **(A)** Principal component analysis (PCA) of high-variance CpGs showing the variability between biological samples and similarities between the replicates (the replicates are indicated as R1 and R2). **(B)** Density plots showing the distribution of the percent DNA CpG methylation in HSPCs of 4-month-old *tet2*^*m/m*^ and 4-month-old *tet2*^*wt/wt*^ fish. The difference between the distribution curves was calculated using the Wilcoxon rank-sum test. **(C)** The percentage differences in the methylation of cytosines within the promoter regions (500 bp to +250 bp upstream and downstream of the transcription initiation site) of annotated genes from 4-month-old *tet2*^*m/m*^ fish compared with 4-month-old *tet2*^*wt/wt*^ fish. The percentage difference in the methylation status of CpG sites in each promoter region is plotted on the *x*-axis and the negative logarithm of the adjusted *p*-value is on the *y*-axis. The promoters with an absolute change greater than 15% and an adjusted *p*-value less than 0.05 are colored. Genes that were hypermethylated in the 4-month-old *tet2*^*m/m*^ fish are colored in orange and those that were hypomethylated are colored in blue. **(D)** Gene set enrichment analysis of hypermethylated gene promoters (human orthologs) against the transcription factor target ChEA 2022 database using EnrichR. The five most significant pathways are shown. The *x*-axis indicates the negative logarithm of the *p*-value from the enrichment results. **(E)** Gene set enrichment analysis of hypomethylated gene promoters (human orthologs) against the transcription factor target (ChEA 2022) database using EnrichR, as in panel **(C)** The five most significant pathways were plotted. The *x*-axis shows the negative logarithm of the *p*-value from the enrichment results. **(F)** The percentage differences in the methylation of cytosines within the CpG island regions in 4-month-old *tet2*^*m/m*^ fish compared with 4-month-old *tet2*^*wt/wt*^ fish. The percentage difference in the methylation status of CpG sites in each CpG island region is plotted on the *x*-axis and the negative logarithm of the adjusted *p*-value is on the *y*-axis. The CpG islands with an absolute change greater than 15% and an adjusted *p*-value less than 0.05 are colored. The CpG islands that were hypermethylated in the 4-month-old *tet2*^*m/m*^ fish are colored in orange and those that were hypomethylated are colored in blue.

To examine the changes in DNA methylation and gene expression with aging of hematopoietic progenitors in normal and *tet2*-mutant zebrafish, we compared the DNA methylation status of CpG nucleotides across the zebrafish genome and gene expression by RNA-seq in the fluorescence-activated cell sorted (FACS) kidney marrow progenitor cells (HSPCs) of 4-month-old zebrafish with those of 15-month-old zebrafish (Figure 1). For the analysis of the DNA methylation status, we employed ERRBS, and RNA-seq was conducted using poly(A)-enriched RNA (see Methods, Supplementary Table 1 for sequencing statistics). PCA of the DNA methylation results separated the results based on the biological replicated pools, indicating the segregation of the biological replicates for each genotype at 4 and 15 months of age (Figure 2A), which supports a distinct DNA CpG methylation pattern in each of the genetic subtypes in fish at maturity (i.e., 4 months of age) and old age (i.e., 15 months of age).

### Loss of *tet2* associates with gain of DNA CpG methylation in HSPCs of 4-month-old fish

We identified a higher density of 75%–100% DNA CpG methylation in 4-month-old *tet2*^*m/m*^ fish than in 4-month-old *tet2*^*wt/wt*^ fish (Wilcoxon rank-sum test *p* < 0.05; Figure 2B), which was consistent with the role of *tet2* in catalyzing the removal of CpG methylation (20). To examine the differences in DNA methylation at the level of specific gene promoters upon *tet2* loss, we compared the overall methylation levels of promoter regions in 4-month-old *tet2*^*m/m*^ fish with those in 4-month-old tet2^*wt/wt*^ fish, as visualized in the volcano plot shown in Figure 2C. Of the 4,096 promoter regions analyzed, 98 were significantly hypermethylated in 4-month-old *tet2*^*m/m*^ fish, and 20 were hypomethylated [Figure 2C and Supplementary Table 2A; methylation difference of at least 15% and statistical significance (q-value) of less than 0.05].

We also examined the genes and pathways that were affected by differential CpG methylation in *tet2*-mutant hematopoietic progenitor cells. We conducted gene set enrichment analysis (GSEA) to identify transcription factor gene targets with promoters that were significantly hypermethylated or hypomethylated in the HSPCs of 4-month-old *tet2*^*m/m*^ fish compared with those of *tet2*^wt/wt^ fish, based on the CHEA Transcription Factor Target Dataset (21, 22). Of the 98 hypermethylated promoters in 4-month-old *tet2*^*m/m*^ fish, 28 were predicted to be targets of *SUZ12*, a component of the polycomb repressive complex 2 (PRC2), which mediates H3K27 trimethylation (H3K27me3) (23). Another 21 gene promoters with increased methylation were predicted to be targets of the metal-response-element-binding transcription factor 2 (*MTF2*) (*p*-value < 0.05). *MTF2* is a metal-response-element-binding transcription factor that recruits PRC2 to specific genes (24). Furthermore, 19 of the hypermethylated genes are common targets of *SUZ12* and *MTF2*, including the transcription factors *NKX-2, DBMX1, TSC22D3*, and *NR2F2.* Therefore, the pathways affected by increased methylation due to *tet2* loss involve both the recruitment and functional activity of PRC2 (Figure 2D, Supplementary Table 2B). Hypomethylated gene promoters in 4-month-old *tet2*^*m/m*^ fish were the targets of the master transcription factors *MYB, FOXM1, SCL (or TAL1)*, and *KLF5*, which are involved in the control of cell proliferation (Figure 2E, Supplementary Table 2C) (25, 26). Each of these master transcription factors is upregulated in cancers of different types.

We also assessed differences in the methylation levels of CpG islands (CGIs) between 4-month-old *tet2*^*m/m*^ fish and 4-month-old *tet2*^*wt/wt*^ fish. Of the 4,350 CpG islands assessed, 97 were hypermethylated and 15 were hypomethylated in 4-month-old *tet2*^*m/m*^ fish, similar to the pattern of promoter methylation (Figure 2F, Supplementary Table 2D). Notably, nine of the CpG islands were within gene promoter regions and showed a consistent pattern of differential methylation as the promoter region in 4-month-old *tet2*^*m/m*^ fish (Supplementary Table 2D).

### *tet2*^*m/m*^ HSPCs acquire a distinct DNA methylation signature during aging

**FIGURE 3:**
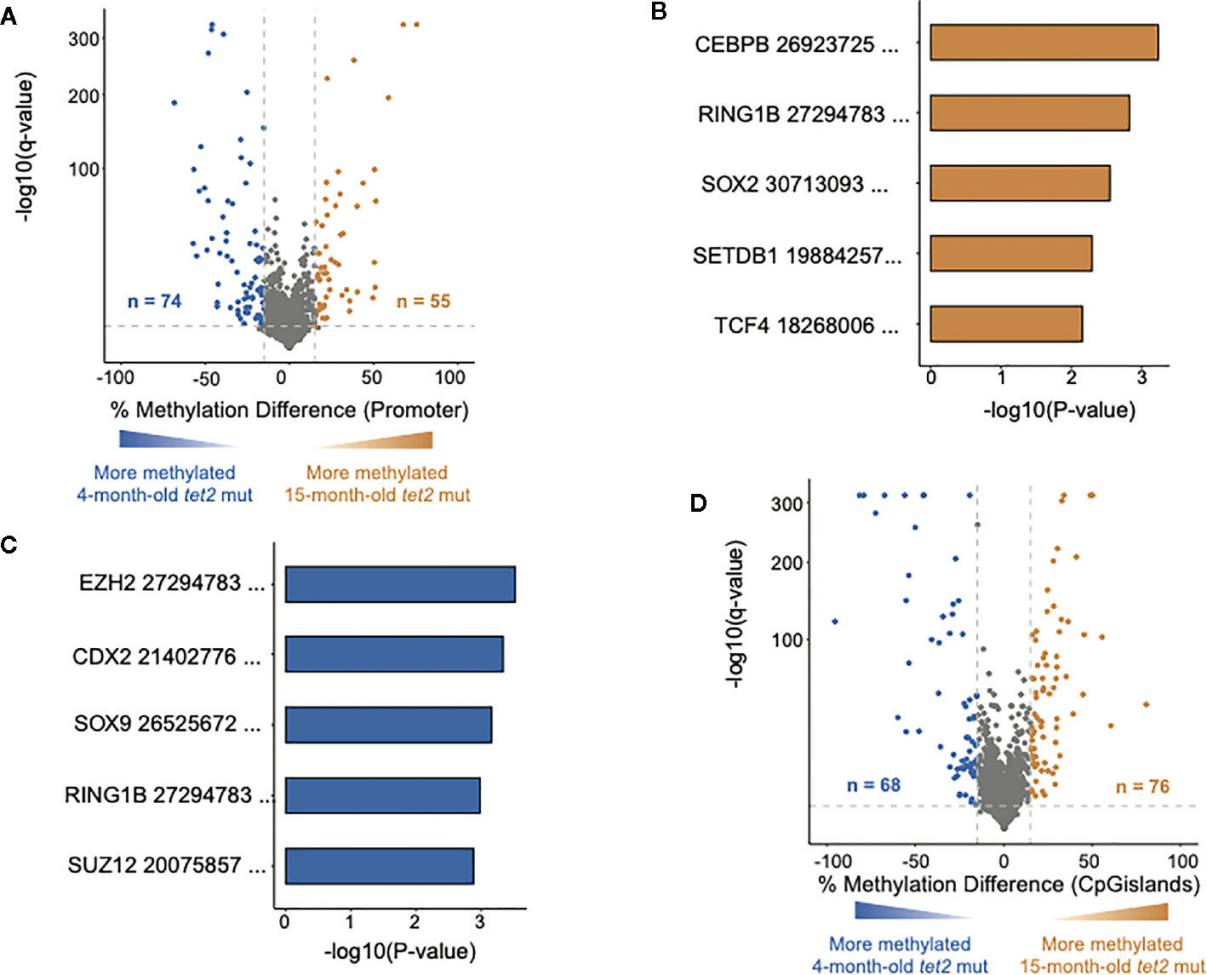
DNA methylation changes in HSPCs of aging *tet2*^*m/m*^ fish. **(A)** The percentage differences in the methylation of cytosines within the promoter regions (500 bp to +250 bp upstream and downstream of the transcription initiation site) of annotated genes from 15-month-old *tet2*^*m/m*^ fish compared with 4-month-old *tet2*^*m/m*^ fish. The percentage difference in the methylation status of each promoter region is plotted on the *x*-axis and the negative logarithm of the adjusted *p*-value is on the *y*-axis. The regions with an absolute change greater than 15% and an adjusted *p*-value less than 0.05 are colored. The genes that were hypermethylated in the 15-month-old *tet2*^*m/m*^ fish are colored in orange and those that were hypomethylated are colored in blue. **(B)** Gene set enrichment analysis of hypermethylated gene promoters (human orthologs) against the ChEA 2022 database using EnrichR. The top five significant pathways were plotted. The *x*-axis indicates the negative logarithm of the *p*-value from the enrichment results. The *y*-axis represents individual pathways. **(C)** Gene set enrichment analysis of hypomethylated gene promoters (human orthologs) against the transcription factor target database (ChEA 2022) using EnrichR. The top five significant pathways were plotted. The *x*-axis indicates the negative logarithm of the *p*-value from the enrichment results. The *y*-axis indicates the individual pathways. **(D)** The percentage differences in the methylation of cytosines within the CpG island regions in 15-month-old *tet2*^*m/m*^ fish compared with 4-month-old *tet2*^*m/m*^ fish. The percentage difference in methylation status of CpG sites in each CpG island region is plotted on the *x*-axis and the negative logarithm of the adjusted *p*-value is on the *y*-axis. The CpG islands with an absolute change greater than 15% and an adjusted *p*-value less than 0.05 are colored. The CpG islands that were hypermethylated in the 4-month-old *tet2*^*m/m*^ fish are colored in orange and those that were hypomethylated are colored in blue.

We next compared the DNA CpG methylation patterns in the gene promoter regions of 15-month-old *tet2*^*m/m*^ fish to those of 4-month-old *tet2*^*m/m*^ fish (Figure 3A). Of the 3,873 promoters analyzed, we found that 55 were significantly hypermethylated and 74 were significantly hypomethylated (Figure 3A, Supplementary Table 3A) at 15 months. We conducted GSEA to identify the transcription factor gene targets with promoters that had acquired significant hypermethylation or hypomethylation at 15 months, based on the CHEA Transcription Factor Target Dataset (18, 22). Hypermethylated gene promoters were identified to be targets of *CEBPB*, *RING1B*, *SOX2*, *SETDB1*, and *TCF4* (Figure 3B, Supplementary Table 3B). In addition, hypomethylated gene promoters were identified to be targets of *EZH2*, *CDX2*, *SOX9*, *RING1B*, and *SUZ12* (Figure 3C, Supplementary Table 3C). In addition to promoters, we also identified changes in DNA methylation in CGIs (Figure 3D, Supplementary Figure 3D). Of the 4,336 CGIs analyzed, we identified 76 that were significantly hypermethylated and 68 that were significantly hypomethylated in the HSPCs of 15-month-old *tet2*^*m/m*^ fish compared with 4-month-old *tet2*^*m/m*^ fish.

We then assessed if the changes in the DNA CpG methylation acquired by HSPCs in 15-month-old *tet2*^*m/m*^ fish were a continuation of the differences identified between 4-month-old *tet2*^*m/m*^ and 4-month-old *tet2*^*wt/wt*^ fish (Supplementary Figure 1). We found that 89 of the gene promoters that were unchanged (or not significantly differentially methylated) in the 4-month-old fish were newly differentially methylated in the 15-month-old *tet2*^*m/m*^ fish compared with the 4-month-old *tet2*^*m/m*^ fish (Supplementary Figure 1). Interestingly, the new DMRs arising in 15-month-old *tet2*^*m/m*^ fish were approximately evenly divided between significant hypermethylation and hypomethylation. Among the 44 newly hypermethylated gene promoters, 15 were predicted to be either *CEBPB or RING1B* targets, whereas the targets of the 45 newly hypomethylated gene promoters included *CDX2*, *YY1*, *CEBPB*, *ERA*, and *CREB1* (Supplementary Figure 1 and Supplementary Table 4).

Furthermore, 66 of the gene promoters that were hypermethylated in the HSPCs of 4-month-old *tet2*^*m/m*^ fish compared with those of 4-month-old *tet2*^*wt/wt*^ fish were not differentially methylated in the 15-month-old *tet2*^*m/m*^ compared with the 4-month-old *tet2*^*m/m*^ fish.

Twenty-eight promoters were hypermethylated in the HSPCs of 4-month-old *tet2*^*m/m*^ compared with those of 4-month-old *tet2*^*wt/wt*^ fish; these then lost methylation in 15-month-old *tet2*^*m/m*^ fish, whereas seven hypomethylated promoters in the HSPCs of 4-month-old *tet2*^*m/m*^ fish, compared with 4-month-old *tet2*^*wt/wt*^ fish, gained methylation in the HSPCs of 15-month-old *tet2*^*m/m*^ fish. Interestingly, the promoters of *TSC22D3* and *NKX2–5*, which were hypermethylated in 4-month-old *tet2*^*m/m*^ fish, become hypomethylated in 15-month-old *tet2*^*m/m*^ fish.

**FIGURE 4:**
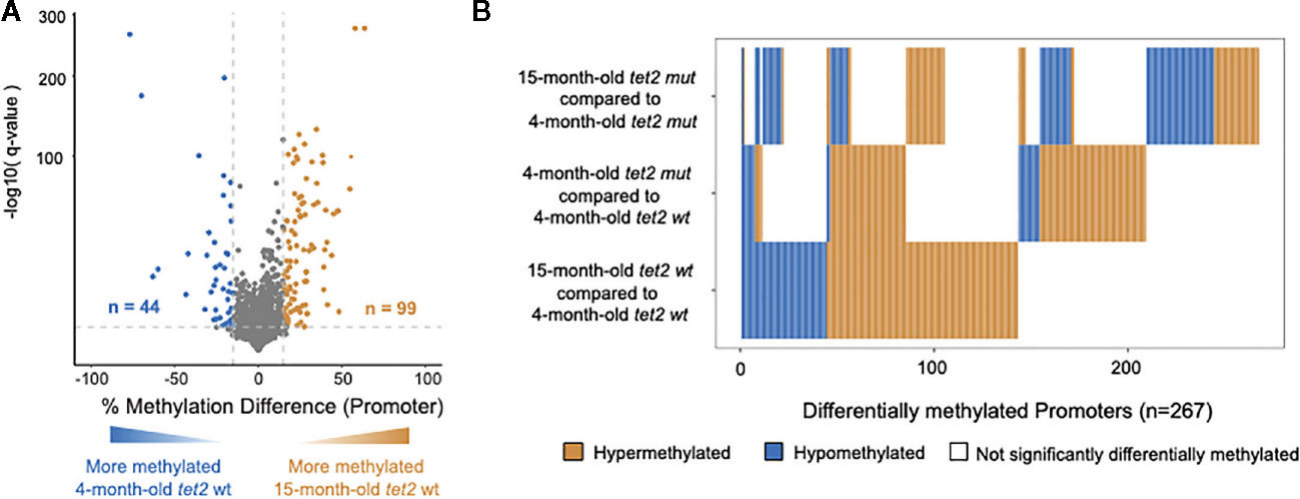
DNA methylation changes in HSPCs of aging *tet2*^*wt/wt*^ fish. **(A)** The percentage differences in the methylation of cytosines within the promoter regions (500 bp to +250 bp upstream and downstream of the transcription initiation site). HSPCs from 15-month-old *tet2*^*wt/wt*^ fish were compared with those of 4-month-old *tet2*^*wt/wt*^ fish, with the latter as the baseline. The percentage difference in the methylation status of CpG sites in each promoter region is plotted on the *x*-axis and the negative logarithm of the adjusted *p*-value is on the *y*-axis. The regions with an absolute change greater than 15% and an adjusted *p*-value less than 0.05 are colored. The genes that were hypermethylated in the 15-month-old *tet2*^*wt/wt*^ fish are colored in orange and those that were hypomethylated are colored in blue. **(B)** Horizontal bar plot comparing the methylation status of significantly differentially methylated promoters identified in 15-month-old *tet2*^*m/m*^ fish compared with 4-month-old *tet2*^*m/m*^ fish (top); 4-month-old *tet2*^*m/m*^ fish compared with 4-month-old *tet2*^*wt/wt*^ fish (middle); and 15-month-old *tet2*^*wt/wt*^ fish compared with 4-month-old *tet2*^*wt/wt*^ fish (bottom). Orange represents > 15% hypermethylation, blue represents > 15% hypomethylation, and white represents no differential methylation.

We next sought to determine if the DNA CpG methylation changes identified in HSPCs between 4- and 15-month-old *tet2*^*m/m*^ fish were distinct from those observed during the normal aging process. We compared the DNA CpG methylation patterns in the gene promoter regions of HSPCs of 15-month-old *tet2*^*wt/wt*^ fish to those of the 4-month-old *tet2*^*wt/wt*^ fish (Figure 4A, Supplementary Table 5A). Of the 4,145 promoter regions analyzed, 99 promoters were hypermethylated and 44 were hypomethylated in the 15-month-old *tet2*^*wt/wt*^ fish (Figure 4A, and bottom row Figure 4B). Interestingly, most of these changes affect different genes than those that differed between the 4-month-old *tet2*^*m/m*^ fish and 4-month-old *tet2*^*wt/wt*^ fish (middle row, Figure 4B). Of the promoters identified as different between the 15-month-old *tet2*^*m/m*^ fish and 4-month-old *tet2*^*m/m*^ fish (top row, Figure 4B), most were different from the promoters that were differentially methylated during normal aging (bottom row, Figure 4B). Thus, of the small percentage of promoters with altered levels DNA methylation during normal aging, most of these alterations appear to depend on *tet2* because they are lost in *tet2*^*m/m*^ HSPCs (Supplementary Table 5B).

### *tet2*^*m/m*^ fish acquire aberrant DNA CpG promoter methylation, which overlaps with changes previously reported in human MDS specimens

**FIGURE 5:**
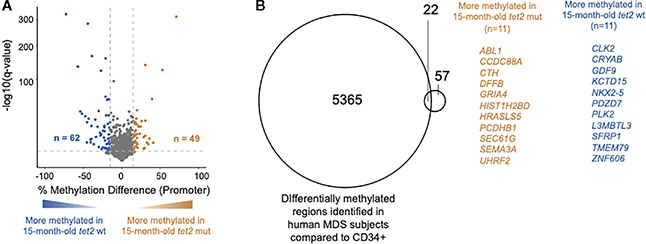
Overlap of DNA CpG promoter methylation in *tet2*^*m/m*^ fish with changes previously reported in human MDS specimens. **(A)** The percentage differences in the methylation of cytosines within the promoter regions (500 bp to +250 bp upstream and downstream of the transcription initiation site) in the HSPCs of 15-month-old *tet2*^*m/m*^ fish compared with those 15-month-old *tet2*^*wt/wt*^ fish, with the latter as the baseline. The percentage difference in the methylation status of the CpG sites in each promoter region is plotted on the *x*-axis and the negative logarithm of the adjusted *p*-value is on the *y*-axis. The regions with an absolute change greater than 15% and an adjusted *p*-value less than 0.05 are colored. The genes that were hypermethylated in the 15-month-old *tet2*^*m/m*^ fish are colored in orange and those that were hypomethylated are colored in blue. **(B)** Overlap of differentially methylated promoters identified in Figure 5A with those identified in human MDS specimens. Common genes are highlighted—genes in orange are hypermethylated in the HSPCs of 15-month-old *tet2*^*m/m*^ fish and those in blue are hypomethylated in the HSPCs of 15-month-old *tet2*^*m/m*^ fish.

We have previously shown that *tet2*-mutant fish develop trilineage dysplasia by 15 months of age, which progresses over the next 7 months to anemia, thus fulfilling the diagnostic criteria of MDS (27). The HSPCs of 15-month-old zebrafish are thus equivalent to the *tet2* mutant “pre-MDS” HSPCs in humans. A previous study assessed for the effect of *tet2* mutations in CD34^+^ HSPCs from MDS patients with age-matched controls (27). To determine if the DNA CpG methylation changes observed in the *tet2* fish model recapitulate the observations in humans, we compared DNA CpG methylation in gene promoter regions of 15-month-old *tet2*^*m/m*^ to the 15-month-old *tet2*^*wt/wt*^ fish (Figure 5A). Of the 3,929 promoters analyzed, we identified 49 hypermethylated and 62 hypomethylated in the 15-month-old old *tet2*^*m/m*^ fish (Figure 5A, Supplementary Table 6). The small number of differentially methylated promoters in HSPCs of 15-month-old *tet2*^*m/m*^ fish overall compared with that in human MDS patient cells presumably reflects the less-complete sequencing coverage of the zebrafish genome and the fact that 15-month-old fish did not yet have MDS, which they would not develop for another 7 months (i.e., at 22 months of age). Nevertheless, 19.8% (*n* = 22) of the significantly differentially methylated gene promoters in 15-month-old zebrafish HSPCs overlapped with published differentially methylated promoters of human orthologs in MDS patients compared with those in age-matched control individuals (Figure 5B). These genes included *NKX2–5*, which is predicted to regulate aberrant gene expression in human MDS (28) and *CLK2*, a proposed oncogene in breast cancer (29); the promoters of both of these genes were hypomethylated in 15-month-old *tet2*^*m/m*^ fish compared with 15-month-old *tet2*^*wt/wt*^ fish.

### Loss of *tet2* in 4-month-old fish causes dysregulation of genes involved in interferon signaling and heme metabolism

**FIGURE 6:**
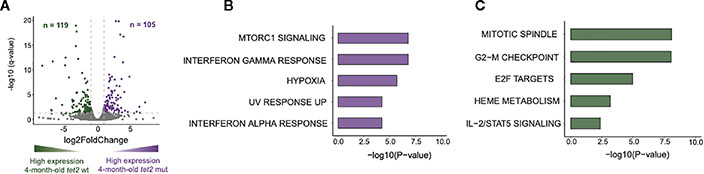
Loss of *tet2* associated with changes in expression of genes associated with inflammation, metabolism, and the cell cycle in 4-month HSPCs. **(A)** Volcano plot representing the differentially expressed genes identified from the bulk-RNAseq sequencing data. Differentially expressed genes were identified between 4-month-old *tet2*^*m/m*^ and 4-month-old *tet2*^*wt/wt*^ HSPCs, with 4-month-old *tet2*^*wt/wt*^ as the baseline. The genes with an absolute log2-fold change greater than 1 and an adjusted *p*-value less than 0.05 are colored. The upregulated genes in the 4-month-old *tet2*^*m/m*^ fish are colored in violet and the downregulated genes in the 4-month-old *tet2*^*wt/wt*^ fish are colored in dark green. **(B)** Pathway enrichment analysis of the upregulated genes against the hallmark database was conducted using EnrichR. The top five significant pathways (*p* < 0.05) were plotted. The *x*-axis indicates the negative logarithm of the *p*-value from the enrichment results. The *y*-axis represents individual pathways. **(C)** Pathway enrichment analysis of the downregulated genes against the hallmark database was conducted using EnrichR. The top five significant pathways were plotted. The *x*-axis indicates the negative logarithm of the *p*-value from the enrichment results. The *y*-axis represents individual pathways.

**FIGURE 7:**
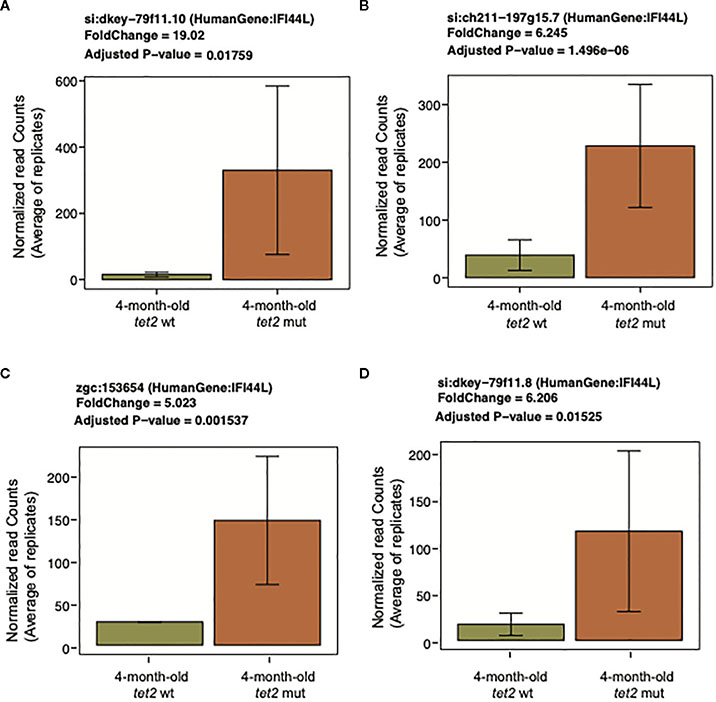
Upregulation of interferon-induced protein 44-like (*IFI44L*). Bar plots representing the normalized expression values averaged across replicates on the *y*-axis and 4-month-old *tet2*^*wt/wt*^ fish and *tet2*^*m/m*^ fish on the *x*-axis. The error bars are calculated as mean expression ± standard deviation. **(A–D)** represent different orthologs of this gene in zebrafish.

We next determined the gene expression pathways that were either upregulated or downregulated in *tet2*^*m/m*^ HSPCs compared with *tet2*^*wt/wt*^ HSPCs in 4-month-old fish. We first identified differentially expressed genes (DEGs) in the 4-month-old *tet2*^*m/m*^ and 4-month-old *tet2*^*wt/wt*^ fish (Figure 6A, Supplementary Table 7A). We identified 105 upregulated genes and 119 downregulated genes (absolute log2-fold change > 1 and q-value < 0.05; Figure 6A). Pathway enrichment of the human orthologs of these zebrafish genes identified several dysregulated pathways within the hallmark gene sets from the Molecular Signature Database (MSigDB) (30). Upregulated genes were enriched in interferon alpha and gamma pathway genes and genes controlling MTORC1 signaling (Figure 6B, Supplementary Table 7B). Interferon-induced protein 44-like gene (*ifi44l*) was included in genesets for both interferon alpha and gamma pathways. Interestingly, each of the four orthologs of *ifi44l* in the zebrafish was upregulated by 5- to 19-fold in *tet2*-mutant zebrafish HSPCs compared with wild-type zebrafish HSPCs, as illustrated in Figure 7. The other upregulated genes in *tet2*-mutant zebrafish HSPCs in the interferon alpha and gamma pathways are shown in Supplementary Figure 2. In addition, the relative expression of representative upregulated genes in the mTORC1 signaling pathway is plotted in Supplementary Figure 3.

**FIGURE 8:**
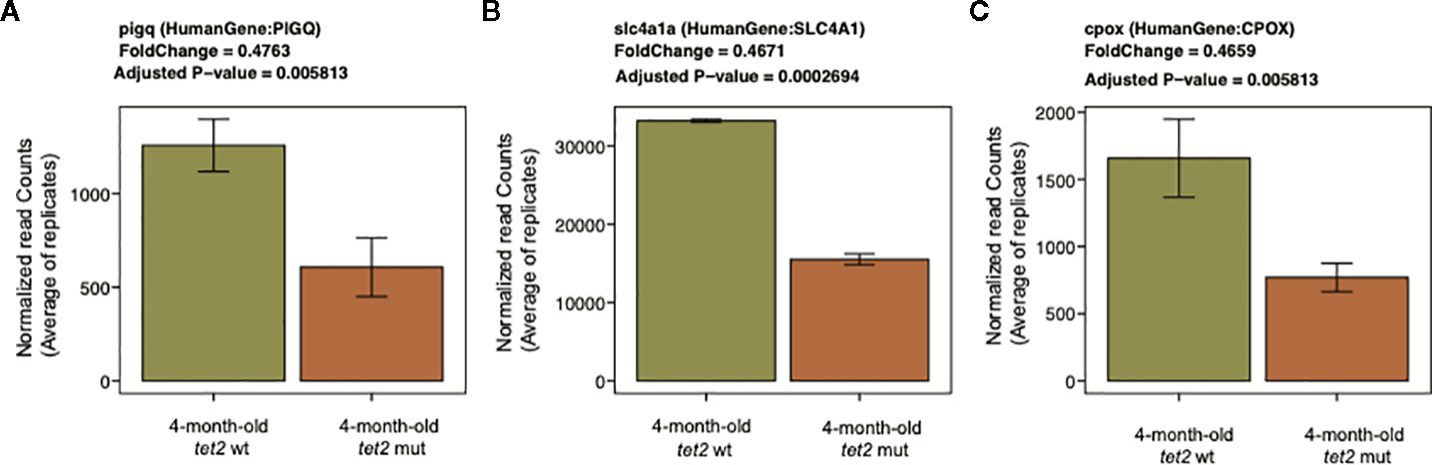
Representative downregulated genes associated with the heme metabolism pathway. Bar plots representing the normalized expression values averaged across replicates on the *y*-axis and 4-month-old *tet2*^*wt/wt*^ fish and *tet2*^*m/m*^ fish on the *x*-axis. The error bars are calculated as mean expression ± standard deviation. PIGQ, Phosphatidylinositol Glycan Anchor Biosynthesis Class Q; SLC4A1, Solute Carrier Family 4 Member 1; CPOX, Coproporphyrinogen Oxidase.

The genes in pathways that were downregulated in tet2-mutant HSPCs include *pqpig*, *slc4a1*, and *cpox*, which are associated with heme metabolism (Figure 8, Supplementary Table 7C). The downregulation of genes involved in heme metabolism in *tet2*-mutant zebrafish HSPCs is in line with the finding that the cell type found to be significantly decreased in the peripheral blood was the erythrocyte. Furthermore, by 24 months of age, most of the *tet2* homozygous mutant fish tested were found to have anemia (7). Downregulated genes were also enriched for the E2F targets and G2M checkpoint (Supplementary Figure 4) and interleukin 2 (IL2)/signal transducer and activator of transcription 5 (STAT5) signaling (Supplementary Figure 5). It will be important in future studies to examine protein expression by Western blotting to verify that the levels of differences of the expressed proteins correspond to the differences of the RNA expression values.

**FIGURE 9:**
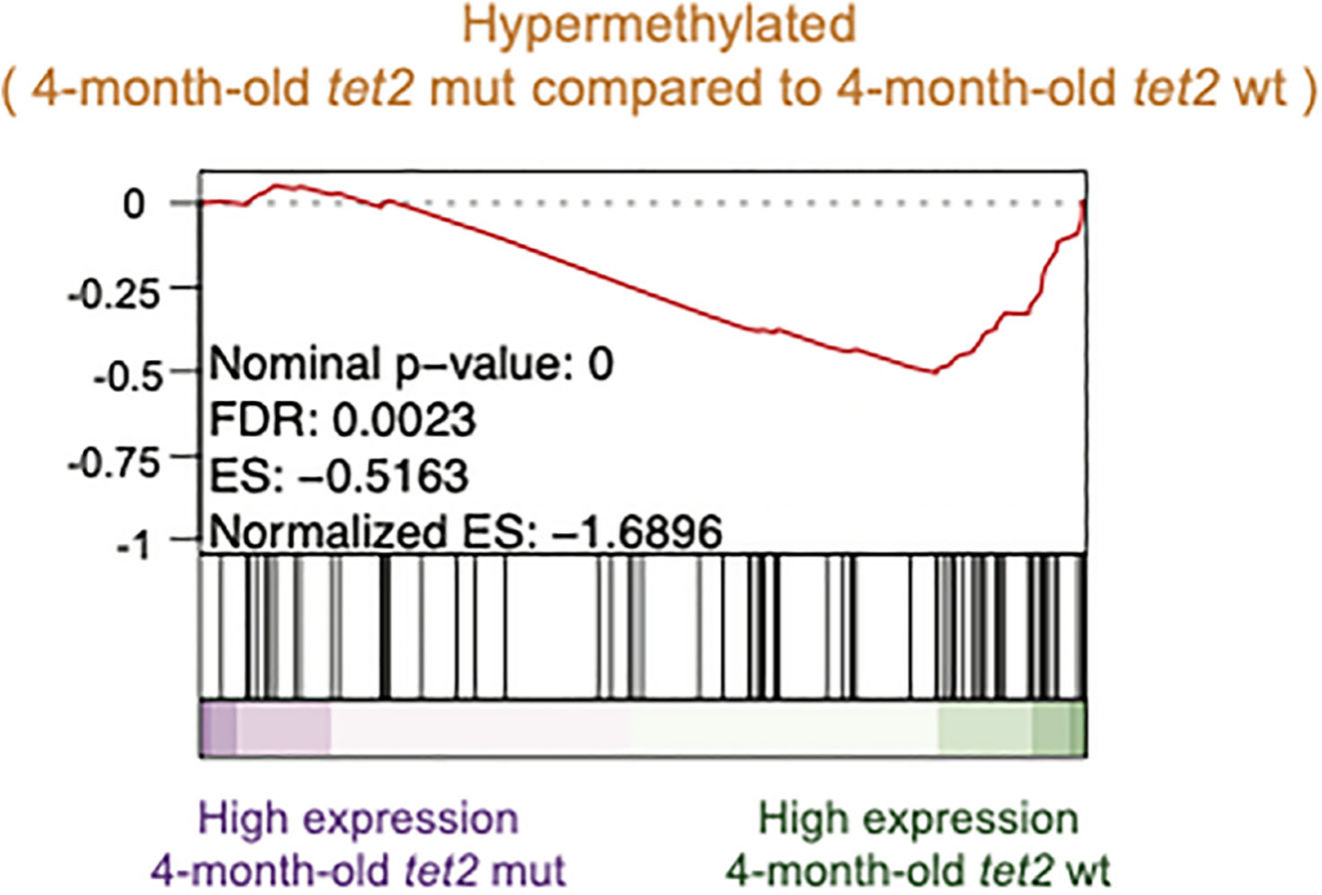
Negative association between hypermethylated gene promoters and gene expression changes observed in 4-month-old *tet2*^*m/m*^ fish compared with 4-month-old *tet2*^*wt/wt*^ fish. Gene set enrichment analysis of hypermethylated promoters identified in Figure 2A against the ranked list of genes (sorted from the highest fold change value to the lowest) identified from Figure 6A.

Finally, we assessed the possible correlation between the gene promoters subject to differential methylation and the pattern of gene expression using GSEA (30). We found a negative association between hypermethylated gene promoters and gene expression changes observed in 4-month-old *tet2*^*m/m*^ fish compared with 4-month-old *tet2*^*wt/wt*^ fish (Figure 9).

## Discussion

In this study, we examined the CpG methylation levels of gene promoters and CpG islands of *tet2*-mutant and wild-type HSPCs in zebrafish at 4 months of age, by which point the fish had reached reproductive age, and at 15 months of age, to analyze age-related changes that occur after maturity. We found that at 4 months of age, five times more gene promoters and CpG islands were hypermethylated in *tet2*-mutant fish compared with wild-type zebrafish than were hypomethylated (Figure 2C), consistent with the known function of *tet2* in catalyzing the removal of methylation from CpG sites in the genome. Interestingly, the hypermethylation was found at promoters of the targets of the polycomb repressive complex 2 (PRC2), which includes SUZ12 and MTF2, known to help recruit PRC2 to its targets in DNA (Figure 2). The PRC2 complex plays an important role in histone trimethylation for the establishment of H3K27me3, which is associated with the repression of gene expression (31).

As noted, the increased proportion of hypermethylated compared with hypomethylated genes in HSPCs at 4 months of age is consistent with the known role of *tet2* in catalyzing the removal of aberrant CpGs that arise during embryogenesis (32). However, in our study of the HSPCs of 15-month-old fish, we observed an unexplained reversal of this trend as the *tet2*-mutant fish matured from 4 months to 15 months of age. In contrast to the predominate hypermethylation of gene promoter CpGs in the 4-month-old *tet2*-mutant fish compared with wild-type HSPCs (Figure 2C), in the 15-month-old *tet2*-mutant fish compared with wild-type HSPCs, we observed an approximately 1.5-fold greater representation of hypomethylated than hypermethylated promoters (Figure 5A). To understand this unexpected trend toward promoter hypomethylation in *tet2*-mutant HSPCs as fish age from 4 months to 15 months, we examined the successive changes with age in *tet2*-mutant fish compared with wild-type fish (Figure 4B). This comparison demonstrates that the change in the promoter DNA methylation pattern does not represent a smooth progression as *tet2*-mutant fish age from 4 to 15 months. This heat map demonstrates that approximately half of the genes that were hypermethylated at 4 months of age remain hypermethylated, whereas a significant proportion become newly hypermethylated or hypomethylated as the *tet2*-mutant fish age to 15 months (right side of the top row of Figure 4B). The basis for these new changes appearing as the fish age and how it relates to the mutational inactivation of *tet2* in these fish is unknown and represents an important question for further study. It is likely that the mechanism driving these changes will prove to be important, because the risk of myeloid malignancy increases with age in both zebrafish and humans with *tet2*-mutant HSPCs.

DNA methylation changes near the transcription start sites have been closely associated with tissue-specific alterations in gene expression (33, 34). Genes with hypermethylated promoters showed overall decreased expression in the HSPCs of 4-month-old fish (Figure 9). Genes that were significantly upregulated in the HSPCs of 4-month-old fish were associated with the interferon alpha and gamma response, which is consistent with recent studies in murine models linking *tet2* mutations to the increased expression of proinflammatory cytokines in *tet2*-mutant myeloid malignancies (35). Further studies investigating the impact of promoter DNA methylation on gene expression *in vivo* in the HSPCs of *tet2* fish compared with wild type fish will benefit from new approaches using single cell RNA-sequencing to separately examine gene expression in different subset of HSPCs in the kidney marrow.

## Supplementary Material

Supplementary Table 1

Supplementary Table 2

Supplementary File

Supplementary Table 3

Supplementary Table 4

Supplementary Table 5

Supplementary Table 6

Supplementary Table 7

## Data Availability

The datasets presented in this study can be found in online repositories. The names of the repository/repositories and accession number(s) can be found below: https://www.ncbi.nlm.nih.gov/geo/query/acc.cgi?acc=GSE238176. Scripts used for analyses and figures’ generation are available here: https://github.com/Yaseswini/TET2_MDS.
