# Supplementary material for: Depletion of tet2 results in age-dependent changes in DNA methylation and gene expression in a zebrafish model of myelodysplastic syndrome
Source: Front Hematol. Author manuscript; Available in PMC 2023 Nov 7. (PMC10629367; doi:10.3389/frhem.2023.1235170)
Supplement: Supplementary Table 1 [file NIHMS1938866-supplement-Supplementary_Table_1.docx]

Supplementary Material

Depletion of *tet2* in zebrafish results in age-dependent changes in DNA methylation and gene expression in zebrafish model of myelodysplastic syndrome

Yaseswini Neelamraju^#^, Evisa Gjini^#^, Sagar Chhangawala, Hao Fan, Shuning He, Chang-Bin Jing, Ashley T Nguyen^,^ Subhash Prajapati, Caroline Sheridan, Yariv Houvras, Ari Melnick, A.Thomas Look^*^, Francine Garrett-Bakelman^*^

^#^ These authors contributed equally

***Correspondence:**

Thomas A Look

thomas_look@dfci.harvard.edu
Francine E. Garrett-Bakelman

[fg5q@uvahealth.org](mailto:fg5q@uvahealth.org)

## Supplementary Tables

**1A)**

**1B)**

**Supplementary Table 1: A)** Sequencing statistics from ERRBS. B) Sequencing statistics from bulk RNA-sequencing.
