# Supplementary material for: Depletion of tet2 results in age-dependent changes in DNA methylation and gene expression in a zebrafish model of myelodysplastic syndrome
Source: Front Hematol. Author manuscript; Available in PMC 2023 Nov 7. (PMC10629367; doi:10.3389/frhem.2023.1235170)
Supplement: Supplementary File [file NIHMS1938866-supplement-Supplementary_File.docx]

Supplementary Material

Depletion of *tet2* in zebrafish results in age-dependent changes in DNA methylation and gene expression in zebrafish model of myelodysplastic syndrome

Yaseswini Neelamraju^#^, Evisa Gjini^#^, Sagar Chhangawala, Hao Fan, Shuning He, Chang-Bin Jing, Ashley T Nguyen^,^ Subhash Prajapati, Caroline Sheridan, Yariv Houvras, Ari Melnick, A.Thomas Look^*^, Francine Garrett-Bakelman^*^

^#^ These authors contributed equally

***Correspondence:**

Thomas A Look

thomas_look@dfci.harvard.edu
Francine E. Garrett-Bakelman

[fg5q@uvahealth.org](mailto:fg5q@uvahealth.org)

# Supplementary Figures

## Supplementary Figures

**Supplementary Figure 1: Comparing differentially** **methylated regions identified between HSPCs of 4-month-old *tet2^m/m^* and 4-month-old *tet2^wt/wt^* to those identified between HSPCs of 15-month-old *tet2^m/m^* and 4-month-old *tet2^m/m^*** Horizontal bar plot comparing the differentially methylated promoters between 15-month-old *tet2^m/m^* and 4-month-old *tet2^m/m^* (top) and 4-month-old *tet2^m/m^* and 4-month-old *tet2^wt/wt^* (bottom). Orange depicts >15% hypermethylation (q < 0.05), blue represents >15% hypomethylation (q<0.05) and white represents no differential methylation

**Supplementary Figure 2: Relative expression of representative significantly upregulated genes associated with interferon alpha and interferon gamma pathways.** Bar plots representing the normalized expression values averaged across replicates on the Y-axis and 4-month-old *tet2^wt/wt^* and *tet2^m/m^* on the X-axis. Error bars are calculated as mean expression +/- standard deviation. MX1= MX Dynamin Like GTPase 1; CASP1 = Caspase 1; STAT1 = Signal Transducer And Activator Of Transcription 1; IFITM3 : Interferon Induced Transmembrane Protein 3.

**Supplementary Figure 3: Relative expression of representative significantly upregulated genes associated with mTORC1 signaling.** Bar plots representing the normalized expression values averaged across replicates on the Y-axis and 4-month-old *tet2^wt/wt^* and *tet2^m/m^* on the X-axis. Error bars are calculated as mean expression +/- standard deviation. NAMPT = Nicotinamide Phosphoribosyltransferase; EGLN3 = Egl-9 Family Hypoxia Inducible Factor 3; CALR = Calreticulin.

**Supplementary Figure 4: Relative expression of representative significantly downregulated genes associated with cell cycle pathways.** Bar plots representing the normalized expression values averaged across replicates on the Y-axis and 4-month-old *tet2^wt/wt^* and *tet2^m/m^* on the X-axis. Error bars are calculated as mean expression +/- standard deviation. NUP107 = Nucleoporin 107; CENPE = Centromere Protein E; HMMR = Hyaluronan Mediated Motility Receptor; ESPL1 = Extra Spindle Pole Bodies Like 1, Separase; SMC4 = Structural Maintenance Of Chromosomes 4.

**Supplementary Figure 5: Relative expression of representative significantly downregulated genes associated with IL2-STAT5 signaling pathway.** Bar plots representing the normalized expression values averaged across replicates on the Y-axis and 4-month-old *tet2^wt/wt^* and *tet2^m/m^* on the X-axis. Error bars are calculated as mean expression +/- standard deviation. S100A1 = S100 Calcium Binding Protein A1; ITIH5 = Inter-Alpha-Trypsin Inhibitor Heavy Chain 5.
